# Pseudomonas aeruginosa: An Unusual Culprit of Left-Sided Native Valve Infective Endocarditis

**DOI:** 10.7759/cureus.56500

**Published:** 2024-03-19

**Authors:** Nazima Khatun, Roy Wang, Ekenedilichukwu N Nnadi, Nimrah Hossain, Suzette Graham-Hill

**Affiliations:** 1 Internal Medicine, State University of New York (SUNY) Downstate Medical Center, Brooklyn, USA; 2 Cardiology, State University of New York (SUNY) Downstate Medical Center, Brooklyn, USA; 3 Cardiology, Kings County Hospital Center, Brooklyn, USA

**Keywords:** transesophageal echocardiography, echocardiography, bacterimia, hemodialysis, endocarditis, pseudomonas, native valve endocarditis, left-sided infective endocarditis, pseudomonas aeuginosa, infective endocarditis

## Abstract

Endocarditis refers to infection or inflammation of the endocardium, and various pathogens can be involved in infective endocarditis (IE). Endocarditis is usually caused by bacteremia in patients with risk factors, including IV drug abuse, indwelling central venous or urinary catheters, recent dental infections, and implantable cardiac devices. *Pseudomonas aeruginosa* (*P. aeruginosa*) is an extremely rare causative organism in IE, predominantly among IV drug users and involving right-sided valves. Left-sided native valve *P. aeruginosa* IE without established risk factors is uncommon. We present a case of a 68-year-old male with no traditional IE risk factors who presented with intermittent fevers. Blood cultures grew *P. aeruginosa*, and transesophageal echocardiography revealed posterior mitral valve vegetation. The patient received broad-spectrum IV antibiotics, which were eventually narrowed down to IV cefepime, guided by culture antimicrobial sensitivities. Although the literature describes various risks for *P. aeruginosa* IE, it can still occur in the absence of traditional predisposing factors. Due to this organism’s rapid resistance acquisition and the complication of septic emboli, an expeditious diagnosis and treatment with antibiotics and/or valve surgery are vital to reducing mortality associated with this entity.

## Introduction

*Pseudomonas aeruginosa* (*P. aeruginosa*) is an aerobic gram-negative bacillus and is an uncommon causative pathogen in infective endocarditis (IE), implicated in approximately 3% of all IE cases. *P. aeruginosa* IE has historically been linked to IV drug use, prosthetic valves, implantable cardiac devices, and hospital-acquired infections [1,2]. Most cases of *P. aeruginosa* IE documented in published reports involve right-sided heart valves and are frequently associated with underlying conditions such as IV drug use, the presence of prosthetic valves, or implanted cardiac devices [2]. Left-sided native valve IE due to *P. aeruginosa* is uncommon. A retrospective review of medical records for acute IE cases by Dawson NL, et al. at the Mayo Clinic across three sites from January 1, 1980, to December 31, 2008, revealed 702 cases of blood cultures positive for *P. aeruginosa* along with a diagnosis of IE, confirming only 4 cases of left-sided native valve endocarditis caused solely by *P. aeruginosa* not linked to IV drug use. This represented 0.6% of all documented acute IE cases at the Mayo Clinic during the study period [3]. Left-sided *P. aeruginosa* IE is an aggressive entity, known for rapidly developing antimicrobial resistance and causing systemic septic emboli. Thus, timely diagnosis and management are imperative for optimal outcomes in *P. aeruginosa* IE [4].

We present a rare case of left-sided native mitral valve *P. aeruginosa* IE in an immunocompetent 68-year-old male with no conventional predisposing risks. This report highlights the importance of early diagnosis and definitive treatment of this high-risk endocarditis, even in the absence of typical risk factors. We also review prior literature on risk factors, diagnosis, management, and outcomes of *P. aeruginosa* IE.

This article was previously presented as a conference abstract at the New York Chapter American College of Physicians (NYACP) in Albany, NY, on May 12, 2023.

## Case presentation

A 68-year-old Hispanic male presented to the ED with a two-day history of fever and chills. At home, his maximum recorded temperature was 101°F (38.3°C).

His past medical history was notable for end-stage renal disease on thrice-weekly hemodialysis via a right-sided arteriovenous graft, aortic valve endocarditis in 1994 with subsequent aortic valve replacement, severe aortic valve insufficiency with a large aortic root pseudoaneurysm status post-new bioprosthetic valve in 2018, coronary artery disease with 60% left anterior descending artery stenosis treated with aspirin 81 mg daily and metoprolol succinate 50 mg daily, heart failure with preserved ejection fraction treated with furosemide 20 mg daily, and hypertension treated with nifedipine extended-release 60 mg daily.

On initial evaluation, he was afebrile with a blood pressure of 125/69 mm Hg, oxygen saturation of 99% on room air, a respiratory rate of 18 breaths per minute, and a regular heart rate and rhythm with normal S1 and S2 sounds. Cardiac examination revealed a systolic murmur, and there was trace bilateral pitting edema upon extremity inspection. The patient denied any history of IV drug abuse. A dental evaluation ruled out any dental abscesses. Initial laboratory tests were significant only for an elevated creatinine level of 8.23 mg/dL (reference range 0.6-1.1 mg/dL) and lactate level of 2.3 mmol/L (reference range 0.5-1.6 mmol/L) without acidosis. Detailed laboratory results are listed in Table 1. Urinalysis was negative for a UTI (Table 2). Respiratory viral testing, including for SARS-CoV-2, was negative.

**Table 1:** Initial laboratory tests results.

Laboratory Tests	Results	Reference Range
Sodium	136	136-145 mmol/L
Potassium	4.4	3.5-4.8 mmol/L
Magnesium	1.85	1.6-2.6 mg/dL
Phosphorus	3.3	2.3-4.7 mg/dL
Chloride	93	98-107 mmol/L
Carbon dioxide	25	22-29 mmol / L
Glucose	93	77-100 mg/dL
Blood urea nitrogen	37	7-20 mg/dL
Creatinine	8.23	0.6-1.1 mg/dL
Estimated glomerular filtration rate (eGFR)	6.5	>= 60 ml/min/1.73m2
Total protein	7.6	6.7-8.6 g/dL
Total bilirubin	0.6	0.2-1.2 mg/dL
Aspartate aminotranferase (AST)	34	5-34 U/L
Alanine transaminase (ALT)	14	0-37 U/L
Alkaline phosphatase	50	40-150 U/L
Calcium	9.6	8.4-10.4 mg/dL
WBC count	6.79	3.80-10.80 K/uL
Hemoglobin	9.9	12-16 g/dL
Hematocrit	31.1	34-45%
Platelet count	130	150-400 K/uL
Venous blood pH	7.36	7.31-7.41
Venous blood partial pressure of carbon dioxide (pCO2)	54	30-50 mmHg
Venous bicarbonate	30	23-28 mmol/L
Lactate	2.3	0.5-1.6 mmol/L

**Table 2:** Urinalysis result. LPF: Low power field; HPF: High power field.

Urinalysis	Results	Reference Ranges
Specific Gravity Urine	1.013	1.005-1.030
Protein Urine	300	Negative mg/dL
Glucose Urine	Negative	Negative mg/dL
Ketones Urine	Negative	Negative mg/dL
Bilirubin Urine	Negative	Negative mg/dL
Blood Urine	Negative	Negative mg/dL
Urobilinogen Urine	1	0.2 - 1.0 EU/dL
Nitrite Urine	Negative	Negative
Leukocyte Esterase	Trace	Negative
Squamous Epithelial	0-5	0 - 5 cells / HPF
WBCs Urine	4.9	0.0 - 5.0 cells / HPF
RBCs Urine	1.5	0.0-5.0 cells / HPF
Bacteria Urine	Few	None seen
pH Urine	>= 9.0	5.0-8.0
Appearance Urine	Clear	Clear
Color Urine	Yellow	Yellow
Cast Urine	5 LPF	0-2 LPF

**Figure 1:**
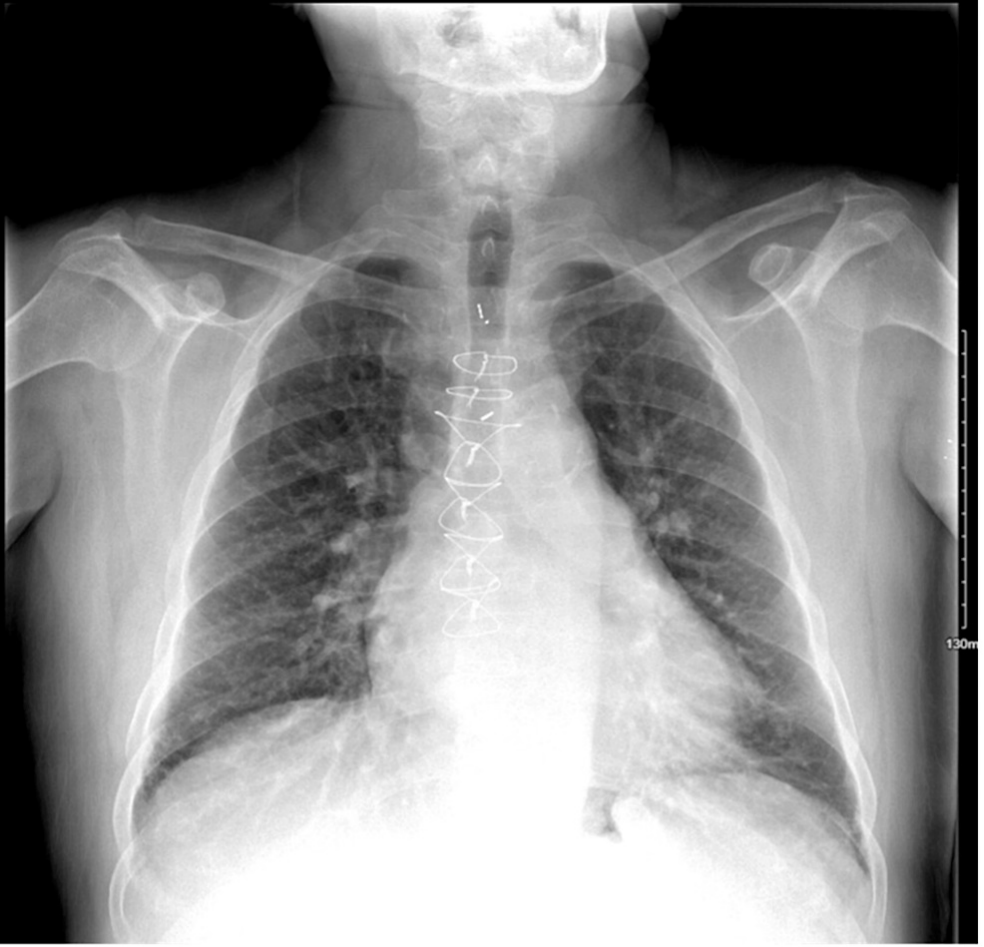
Chest X-ray findings. Chest X-ray without any imaging evidence of obvious lung or cardiac pathology.

An initial ECG exhibited sinus tachycardia and left ventricular hypertrophy. The chest X-ray was unrevealing for any lung or cardiac pathology (Figure 1).

**Figure 2:**
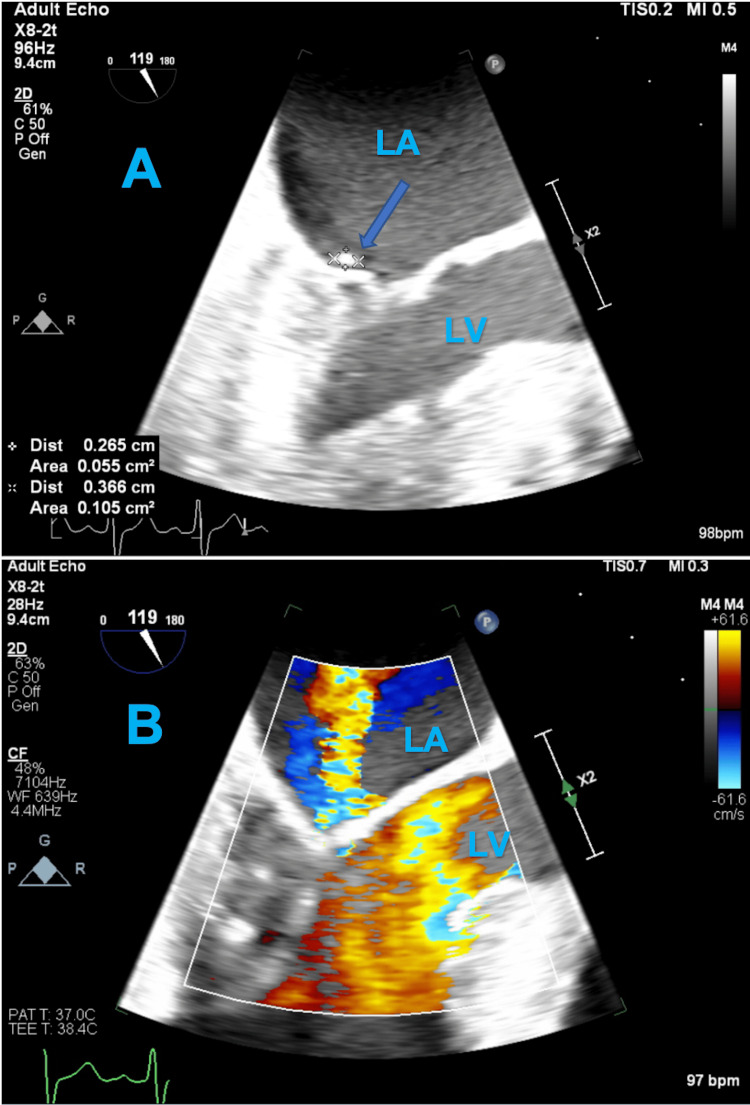
Transesophageal echocardiography (TEE) findings. (A) TEE showing a 0.27 cm x 0.37 cm sessile vegetation on the body of the posterior leaflet of the mitral valve (blue arrow). (B) TEE showing moderate to severe mitral regurgitation. TEE: Transesophageal echocardiography; LA: Left atrium; LV: Left ventricle.

On day 2, transthoracic echocardiography (TTE) was also unrevealing; the ejection fraction was 60%, and the bioprosthetic aortic valve had normal function. No vegetations were noted on any of the valves. On day 6, a transesophageal echocardiogram (TEE) (Figure 2) revealed significant findings: a small sessile vegetation measuring 0.27 cm x 0.37 cm on the body of the posterior leaflet of the mitral valve, a small mitral valve perforation at the tip of the posterior leaflet, and moderate to severe mitral regurgitation. There was no involvement of the prosthetic aortic valve. The left atrium was severely dilated, and the right atrium was moderately dilated on TEE.

In the ER, blood cultures were drawn, and the patient was initially started on broad-spectrum intravenous antibiotics with vancomycin and piperacillin-tazobactam, and admitted to the medicine floors while awaiting blood culture speciation and antimicrobial sensitivities. The patient continued to have episodes of fever, and four sets of initial blood cultures grew *P. aeruginosa*. The patient was switched from piperacillin-tazobactam to cefepime because cefepime has a minimum inhibitory concentration (MIC) of 2 or less, whereas piperacillin-tazobactam has an MIC of 8 or less. Vancomycin was discontinued. A multidisciplinary team, comprising the primary medicine team, cardiology, infectious disease, and social work, was involved in the co-management of the patient. Follow-up blood cultures were negative on two occasions. The infectious diseases team recommended continuing with intravenous cefepime for a total of six weeks. Before discharge, the social worker coordinated the prescription of cefepime with the patient’s hemodialysis center. The patient remained stable on cefepime and, after one week, was discharged with instructions to complete a six-week regimen of IV cefepime during hemodialysis sessions.

## Discussion

*Pseudomonas aeruginosa* is an exceptionally uncommon causative organism implicated in causing IE. Over 95% of *P. aeruginosa*-mediated IE involves right-sided valves, predominantly among intravenous drug users [4]. In contrast, a review of published literature reveals only 36 confirmed reports of left-sided *P. aeruginosa* IE in non-intravenous drug user patients thus far [3,4], affirming the uniqueness of our case.

The predominant risk factor for *P. aeruginosa* IE among published cases in non-intravenous drug user populations was prior cardiac intervention or surgery, accounting for 34% of cases. Other common predisposing factors were genitourinary infections (29%) and hemodialysis dependence (17%) [3]. Our patient had isolated mitral valve involvement, with his prosthetic aortic valve unaffected. Typically, risk factors for Pseudomonal IE in immunocompetent individuals include indwelling bladder catheters, central venous lines, or traumatic and postoperative infections - none of which were present here.

Of relevance, arteriovenous fistula and dialysis catheter infections pose significant risks for hemodialysis patients. In a 15-year (1990-2004) retrospective review, Kamalakannan D et al., demonstrated that patients on chronic hemodialysis, where the predominant vascular access type was a double-lumen catheter (66.7%), are predisposed to IE as a disastrous complication of transient bacteremia, usually attributable to gram-positive organisms but rarely gram-negative bacteria such as *P. aeruginosa* (5.8%) [5]. In essence, while diverse risk factors for *P. aeruginosa* IE are acknowledged, our case represents a constellation without commonly associated predisposing elements - thereby amplifying its rarity and underscoring the need for prompt recognition and treatment of pseudomonal endocarditis even in their absence.

Left-sided IE, in contrast to disease limited to the right heart valves, portends higher risks for abrupt systemic septic embolization and poorer prognosis [6]. Prior studies have reported higher mortality among IE patients manifesting with clinically evident embolic phenomena [7]. A review of the literature reveals only 11 total reported survivors of left-sided pseudomonal endocarditis among non-intravenous drug-using individuals. Notably, 7 of these 11 patients had undergone infected valve replacement surgery as a component of treatment [3], underscoring the role of prompt diagnosis and definitive management, encompassing both antibiotic therapy and consideration for valve replacement surgery where viable.

The paucity of prior documented survivors and the frequent need for surgery reinforce the high-risk nature and rapidly progressive course of left-sided pseudomonal endocarditis in the absence of traditional risk factors like intravenous drug use. Hence, maintaining a high index of suspicion and a low threshold for aggressive management involving infectious disease specialists, cardiologists, and cardiothoracic surgery services is imperative.

## Conclusions

In summary, we present an exceptional case of left-sided native mitral valve *P. aeruginos*a IE in an immunocompetent patient without traditional risk factors. Given the intermittent fevers and a history of valve surgery, we maintained a high suspicion for IE. This case underscores the necessity of prompt recognition and aggressive management, even in the absence of risk factors. Maintaining a low threshold for workup is crucial to confirm an early diagnosis and tailor definitive treatment, considering the rapid progression and poor prognosis of Pseudomonal endocarditis. While traditional risk factors were absent, the patient's hemodialysis and prior valve surgery might have contributed to this atypical case of IE. Prudence dictates the consideration of uncommon potential risks when classic factors are not present.
